# Performance Scores in General Practice: A Comparison between the Clinical versus Medication-Based Approach to Identify Target Populations

**DOI:** 10.1371/journal.pone.0035721

**Published:** 2012-04-20

**Authors:** Olivier Saint-Lary, Philippe Boisnault, Michel Naiditch, Philippe Szidon, Didier Duhot, Yann Bourgueil, Nathalie Pelletier-Fleury

**Affiliations:** 1 Prospere, Paris, France; 2 Département de médecine générale PIFO, Université Versailles Saint-Quentin en Yvelines, Guyancourt, France; 3 Société Française de Médecine générale (SFMG), Issy les Moulineaux, France; 4 Institut de recherche et documentation en économie de la santé (IRDES), Paris, France; 5 CERMES3 - UMR 8211 - INSERM U988, Villejuif, France; Yale University School of Medicine, United States of America

## Abstract

**Context:**

From one country to another, the pay-for-performance mechanisms differ on one significant point: the identification of target populations, that is, populations which serve as a basis for calculating the indicators. The aim of this study was to compare clinical versus medication-based identification of populations of patients with diabetes and hypertension over the age of 50 (for men) or 60 (for women), and any consequences this may have on the calculation of P4P indicators.

**Methods:**

A comparative, retrospective, observational study was carried out with clinical and prescription data from a panel of general practitioners (GPs), the Observatory of General Medicine (OMG) for the year 2007. Two indicators regarding the prescription for statins and aspirin in these populations were calculated.

**Results:**

We analyzed data from 21.690 patients collected by 61 GPs via electronic medical files. Following the clinical-based approach, 2.278 patients were diabetic, 8,271 had hypertension and 1.539 had both against respectively 1.730, 8.511 and 1.304 following the medication-based approach (% agreement = 96%, kappa = 0.69). The main reasons for these differences were: forgetting to code the morbidities in the clinical approach, not taking into account the population of patients who were given life style and diet rules only or taking into account patients for whom morbidities other than hypertension could justify the use of antihypertensive drugs in the medication-based approach. The mean (confidence interval) per doctor was 33.7% (31.5–35.9) for statin indicator and 38.4% (35.4–41.4) for aspirin indicator when the target populations were identified on the basis of clinical criteria whereas they were 37.9% (36.3–39.4) and 43.8% (41.4–46.3) on the basis of treatment criteria.

**Conclusion:**

The two approaches yield very “similar” scores but these scores cover different realities and offer food for thought on the possible usage of these indicators in the framework of P4P programmes.

## Introduction

Over the past few years a set of indicators has been developed to measure the quality of healthcare in the framework of pay for performance (P4P) programmes [1], [2]. The underlying principle is that health practitioners are rewarded for the achievement of certain quality standards in their healthcare delivery, measured against a set of specific indicators. The logic derives from standard economic theory which holds that appropriate external incentives (here, financial) are likely to alter individuals' behaviour and steer it in the required direction [3]. This new mode of remuneration has targeted primary care and especially general practice [4].To date, the most conclusive experiment in this respect has been in the UK where P4P has been implemented since April 2004 with a set of indicators developed in the Quality and Outcomes Framework [5], [6]. Other countries such as the US, Australia, New Zealand and Israel have also adopted this mode of payment [7], [8]. In France the introduction of a system based on a similar principle was proposed in 2009 by the national health insurance fund for employees (*Caisse Nationale d'Assurance Maladie des Travailleurs Salariés (CNAMTS)*) [9], under its P4P programme called Contract for Enhancing the Individual Practices (*Contrats d'Amélioration des Pratiques Individuelles (CAPI)*). This programme is based on a set of 16 indicators. Nine of them have healthcare objectives and are divided into two categories. The first, “screening and prevention”, concerns for example the percentage of a doctor's patients in the 50–74 age-group who have had breast-cancer screening, or the rate of flue vaccination among patients over 65. The second, “treatment of chronic pathologies”, primarily concerns adherence to recommendations concerning diabetes and hypertension. The other seven indicators, under the heading “optimization of prescriptions”, aim at rationalizing healthcare and encouraging the prescription of generic medicines.

From one country to another, the P4P mechanisms differ on one significant point: the identification of target populations, that is, populations which serve as a basis for calculating the indicators. In the UK, target populations are identified on the basis of clinical data collected by practitioners in a standardized way during consultations. For this purpose doctors use medical software meeting compatibility standards and enabling them to transfer their data. This mode of identification is criticized in certain respects, mainly because it allows for misreporting when doctors code data [10], [11], and because the time taken to code data may reduce the time spent listening to the patient [12]. In France, the identification of target populations is based on the medication prescribed by doctors and reimbursed by the compulsory health insurance fund. These data are drawn from the health insurance fund's database which routinely collects information from all patients affiliated with this fund for the reimbursement of their health care. This mode of identification based on reimbursed medication does not require doctors to code data. But already the limits of this mode of identification based on medication have emerged. This is for example because the same medicine may have several indications [13]. To our knowledge, no comparative research of these two modes of identification or of their consequences on the calculation of indicators has been yet undertaken.

We considered it interesting to study these two approaches, drawing on an original database containing both the diagnoses and the prescribed treatments of over 80,000 patients. This database is fed by data collected routinely by a network of volunteer GPs [14]. To this end we chose a target population whose care is subject to good practice recommendations, that is, patients with three cardio-vascular risk factors: age, hypertension and diabetes.

The aim of this study was to compare clinical versus medication - based identification of populations of patients with diabetes and hypertension over the age of 50 (for men) or 60 (for women), and any consequences this may have on the calculation of P4P indicators.

## Materials and Methods

We carried out a comparative, retrospective, observational study.

### General practitioner sampling

Data on patients, diseases and related health problems were drawn from French GPs' electronic medical records. These were accessed via the database which the French Society of General Practice (*Société Française de Médecine Générale (SFMG)*) has been compiling since 1993 in a network of 90 GPs working mainly in solo practices (SFMG-DB). The participants in this network routinely register data in their daily practice. They are largely representative of the French GP population, although a comparison with data from the Ministry of Health shows that doctors working in rural areas were under-represented [15], [16]. We studied the practices of the 61 GPs for whom complete information with regard to diseases and related health problems or prescriptions were available during 2007 i.e. just before the implementation of the CAPI.

### Patient registration

The 61 GPs had cared for a total of 81,052 patients whose age and sex distribution did not differ significantly from that of the population as a whole. For the present data-based study, we only selected patients older than 50 for men and 60 for women, that is, populations which served as a basis for calculating the indicators (see below), which gave us a sample of 21,690 patients.

### Identification using clinical codes

In the SFMG-DB, diseases and related health problems are coded using the Dictionary of Consultation Results (DCR), which has been validated in France [17]. The corresponding codes in the International Classification of Diseases, 10th Revision, Clinical Modifications (ICD-10-CM) are also mentioned. The diseases and related health problems of interest in our study were type 2 diabetes (CR 818/ICD E11) and hypertension (CR 826/ICD I10).

### Identification using medications

In the SFMG-DB, medications are coded according to the Anatomical Therapeutic Chemical (ATC) Classification System (WHO, 2006). We retained two therapeutic categories for diabetes: (i) Insulin: ATC group A10A, and (ii) oral anti-diabetic medics: ATC group A10B and C10A. Five therapeutic categories were retained for hypertension: (i) Adrenergic beta-antagonists: ATC group C07, (ii) diuretics: ATC groupC03, (iii) Calcium channel blockers: ATC group C08, (iv) Angiotensin-converting enzyme inhibitors: ATC group C09A and Angiotensin II Type 1 Receptor Blockers: ATC group C09C, (v) other antihypertensive agents: ATC group C02 (Table 1). ATC codes for acetyl salicylic acid and statins were respectively B01AC06, N02BA01 and C10AA01–C10AA08.

**Table 1:** ATC codes for prescription data.

Indication	Classe ATC	Molecules
Diabète	A10A	human insulin, bovine insulin, pig insulin, insulin asparte, insulin glulisine
	A10B	metformin, glibenclamide, chlorpropamide, tolbutamine, glibornuride, carbutamide, glipizide, gliclazide, glimepiride, acarbose, miglitol, rosiglitazone, pioglitazone, sitagliptine, repaglinide, exanatide
	C10A	benflorex
Hypertension	C02	reserpine, methyldopa, clonidine, guanfacine, tolonidine, moxonidine, rilmenidine, prazosine, urapidil, dihydralazine, minoxidil
	C03	bendroflumethiazide, hydroflumethiazide, hydrochlorothiazide, polythiazide, methyclothiazide, clopamide, chlortalidone, xipamide, indapamide, cicletanine, furosemide, piretanide, tienilique acide, spironolactone, canrenone, amiloride, triamterene
	C07	oxprenolol, pindolol, propranolol, timolol, nadolol, carteolol, tertatolol, penbutolol, metoprolol, atenolol, acebutolol, betaxolol, bisoprolol, celiprolol, nebivolol, labetalol
	C08	amlodipine, felodipine, isradipine, nicardipine, nifedipine, nitrendipine, lacidipine, manidipine, lercanidipine, mibefradil, verapamil, diltiazem
	C09A	captopril, enalapril, lisinopril, perindopril, ramipril, quinapril, benazepril, cilazapril, fosinopril, trandolapril, moexipril, zofenopril, imidapril

### Constitution of target populations according to the two modes of identification

With the medication approach, we first identified two populations within the set of patients over the age of 50 (men) or 60 (women): the “Medication Identified Diabetes” and the “Medication Identified Hypertension” populations. They were constituted only on the basis of the medication prescribed. To be included in the population, a patient had to have received, in the year 2007, at least one prescription for a diabetes treatment (oral or insulin), for the “Medication Identified Diabetes”, and at least one prescription for a hypertension treatment, for the “Medication Identified Hypertension” population. Finally, by cross-comparing the two populations it was possible to obtain the “Medication Identified Diabetes and Hypertension” population corresponding to our target population identified on the basis of treatment.

With the clinical approach, we identified the “Diagnostic Code Identified Diabetes” population and the “Diagnostic Code Identified Hypertension” population, based exclusively on clinical data. To be included in the “Diagnostic Code Identified Diabetes”population, a patient had to have in his/her file, for the year 2007, at least one type-2 diabetes CR, and to be included in the “Diagnostic Code Identified Hypertension” population, at least one hypertension CR. The cross-comparison of the two populations enabled us to obtain the “Diagnostic Code Identified Diabetes and Hypertension” population, that is, our target population identified on the basis of clinical data.

### Analysis of data concordance

We calculated % agreement, and Kappa scores to analyse data concordance between the two approaches.

### Calculation of the P4P indicators

Two indicators defined following the guidelines developed by the “Haute Autorité de Santé” [18] were then calculated according to the two modes of identification:

Statin Indicator = patients (age>50 for men and >60 for women) with diabetes and hypertension, taking statin/patients (age>50 for men and >60 for women) with diabetes and hypertension. For information, the target objective for this indicator in the CAPI framework is 75%.

Aspirin Indicator = patients (age>50 for men and >60 for women) with diabetes and hypertension, taking statin and aspirin/patients (age>50 for men and >60 for women) with diabetes and hypertension taking statin. The target objective for this indicator in the CAPI framework is 65%.

The indicators were calculated individually for the 61 GPs. The results were expressed as mean (IC at 95%).

### Ethics committee

We did not seek ethical approval for this study because in France, there was no need for an ethics committee approval as the data used in this study were collected as part of routine medical practice and also because there was no supplementary data collected and no specific intervention on the patient. However, it should be mentioned that patients all gave their informed consent for the anonymous registration of their clinical data in the SFMG-DB and also for the use of these data for research and this was approved by the Commission Nationale de l'Informatique et des Libertés (CNIL) (approval n° 311668).

## Results

### Target population of diabetic patients according to the two modes of identification

Among 21,690 patients in our sample, 19,412 did not have a diagnostic code for diabetes, whereas 2,278 did; in contrast, 19,960 did not receive a medication for diabetes, whereas 1,730 did (% agreement = 96%, kappa = 0.78) (Table 2). The 666 patients clinically coded as being diabetic but who received no medication for diabetes can be considered as diabetic patients treated exclusively with rules pertaining to life style and diet. This represented 27.8% (666/(1,730+666) of the patients with diabetes in our sample. It should be noted that as treatment data are taken directly from the prescriptions drawn up on computer and delivered to patients, theoretically the doctor could not have forgotten to enter the ATC code. The 118 patients who received a treatment for diabetes without the diabetes CR code being recorded correspond to cases where the code was forgotten, because these were medications which had no indication other than diabetes, except for metformine which is occasionally used to treat polycystic ovarian syndrome but this did not concern any of our patients. The rate of forgetting the code in the clinical approach was 4.9% (118/(2,278+118)).

**Table 2:** Target population of diabetic patients according to the two modes of identification.

	Medication-based identification		
Clinical-based identification	NO	YES	TOTAL
NO	19,294	118	19,412
YES	666	1,612	2,278
TOTAL	19,960	1,730	21,690

### Target population of patients with hypertension according to the two modes of identification

Among 21,690 patients in our sample, 13,419 did not have a diagnostic code for hypertension, whereas 8,271 did; in contrast, 13,179 did not receive a medication for hypertension, whereas 8,511 did (% agreement = 84%, kappa = 0.67) (Table 3). The 1,592 patients with the hypertension CR code being recorded but without a treatment for hypertension can be considered as patients with untreated hypertension, either deliberately because they were following life style and diet rules, or because of delays in starting the treatment. This represented 15.8% (1,592/(8,511+1,592)) of the patients with hypertension in our sample. Some but not all of the 1,832 patients who received a treatment indicated for hypertension, without having the hypertension CR code being recorded, were cases where the code was forgotten. There were also patients for whom morbidity other than hypertension could justify the use of medications that had received a drug approval not only for hypertension but also for another indication. Therefore, these patients did not correspond to cases where the code was forgotten since they did not have hypertension. This concerned 1088 patients, i.e. 12.8% of the population with medications for hypertension (1088/8511), who had at least one morbidity among the following which required a medication of this type (angina pectoris (CR 711/ICD I20.9), heart failure (CR 820/ICD I50.9), atrial fibrillation (CR 819/ICD I48), other cardiac arrhythmias (CR 823/ICD R00.8), isolated leg oedema (CR 223/ICD R60.0), kidney failure (CR 179/ICD N19), migraine disorders (CR 206/ICD G43.9), cirrhosis (CR 838/ICD K74.6), tremor (CR 296/ICD R25.1), hyperthyroidism (CR 604/ICD E05.9), Raynaud's syndrome (CR 7/ICD I73.0) and/or benign prostatic hyperplasia (CR 845/ICD N40)). The remaining 744 could be considered as actually having hypertension and corresponding to a case where the hypertension CR code was forgotten. The rate of forgetting the code in the clinical approach was 8.2% (744/8,271+744)).

**Table 3:** Target population of patients with hypertension according to the two modes of identification.

	Medication-based identification		
Clinical-based identification	NO	YES	TOTAL
NO	11,587	1,832	13,419
YES	1,592	6,679	8,271
TOTAL	13,179	8,511	21,690

### Target population of patients with diabetes and hypertension according to the two modes of identification

Among 21,690 patients in our sample, 20,151 did not have a diagnostic code for diabetes and hypertension, whereas 1,539 did; in contrast, 20,386 did not receive a medication for diabetes and hypertension, whereas 1,304 did (% agreement = 96%, kappa = 0.69) (Table 4). The clinical approach enabled us to identify 531 patients with hypertension and diabetes, not identified by the medication-based method. Conversely, identification on the basis of medications enabled us to identify 296 patients not identified on the basis of clinical criteria. Yet by using the same reasoning as above, 112 of the latter patients must be considered as not having hypertension even though they received treatment for hypertension. Thus, a total of 184 patients were identified as having diabetes and hypertension, based on their medications but not on clinical criteria.

**Table 4:** Target population of patients with diabetes and hypertension according to the two modes of identification.

	Medication-based identification		
Clinical-based identification	NO	YES	TOTAL
NO	19,855	296	20,151
YES	531	1,008	1,539
TOTAL	20,386	1,304	21,690

### Calculation of the P4P indicators

The mean per doctor of statin indicator when the target populations were identified on the basis of clinical criteria was 33.7% (31.5–35.9) whereas it was 37.9% (36.3–39.4) when the target populations were identified on the basis of treatment criteria.

For aspirin indicator, the mean per doctor was 38.4% (35.4–41.4) after clinical identification and 43.8% (41.4–46.3) after medication identification.

**Figure 1:**
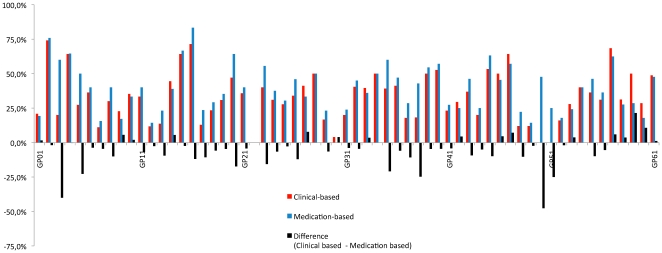
Mean per doctor of statin indicator according to the two modes of identification.

**Figure 2:**
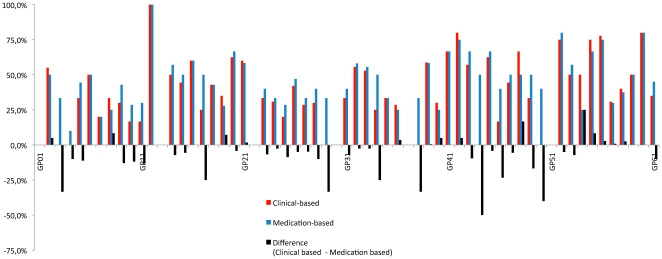
Mean per doctor of aspirin indicator according to the two modes of identification.

Two physicians moved from above to below the expected rates of 65% for the statin indicator when switching from the medication identification to the diagnostic identification. None of them did in the other way. Three physicians moved from above to below the expected rates of 75% for the aspirin indicator when switching from the medication identification to the diagnostic identification, one moved in the other way. (Figures 1 and 2)

## Discussion

The aim of this article was to compare clinical versus medication - based identification of populations of patients with diabetes and hypertension and the effects each of them could have on the calculation of P4P indicators. We showed that there was a quite good degree of data concordance and that the results of the P4P indicators obtained with the two methods differ little in terms of final scores (difference of 4% in favour of the medication-based identification) but that these results correspond to different realities.

We observed that both of these two modes of identification can cause the target populations to be under-estimated, either – to a small extent – due to the doctor forgetting to code the morbidities, in the case of clinical identification, or – to a greater extent – due to the fact that patients whose only treatment is life style and diet rules are not taken into account in medication-based identification. Hence, in both cases the level of the indicator can be over-estimated. In our study, the rate of forgetting to code the morbidities in the clinical approach is 4.9% for diabetes and 8.2% for hypertension. These results are consistent with those already published in the literature assessing the completeness and correctness of computerized general practice medical records where the rate of forgetting to code the morbidities was situated between 5 and 10% [19], [20]. Medication-based identification under-estimates the population of diabetic patients by 27.8% because there are patients who are given life style and diet rules only. The percentage of such patients is higher in our sample than it is in the French literature, where it oscillates between 10% and 15% [21]–[23] without any clear explanation for this. However it goes along with the results of a cross-sectional study of 253 618 patients lead in the UK in 2004 which showed that 31.3% of all patients with type 2 diabetes were being managed with diet only [24]. Concerning patients with hypertension, the medication-based identification under-estimates this population by 15.8% due to patients following lifestyle and diet rules only, and to the well-known therapeutic inertia in hypertension. This rate of patients with untreated hypertension is lower than that reported in a large country-wide study on general practice in France where one third of the 70,000 studied had never received hypertension medication [25]. This rate was also estimated at 27.5% in a US study [26].

The medication mode of identification can on the other hand result in an over-estimation of the target population of patients with hypertension, and consequently in the under-estimation of the level of the indicator, since medications for hypertension may be indicated to treat morbidities other than hypertension. In our study the identification of patients with hypertension on the basis of medication led to the inclusion of 12.8% of patients who did not have hypertension and should not have been included. To our knowledge this has never been underlined before. A perverse effect of this medication-based identification, in the framework of a P4P programme, could be to expose these patients to the risk of being prescribed statins by non scrupulous doctors for the sole purpose of improving their individual scores.

From an economic point of view, if we were in the framework of a P4P programme, the mode of identification would not substantially change doctors' financial remuneration. This is because the final scores obtained look “quite similar” when we compare the clinical and medication-based methods whereas they deviate from the target objectives by over 20%. However, even if we cannot make any statistical comparison between the results obtained with the two methods, when we look at the confidence intervals calculated for the two indicators selected, the medication-based identification method appears in our sample to be more advantageous for doctors. This is probably due to the fact that the diabetic population treated by lifestyle and diet rules only, which is huge in our sample, is not included in this method of calculating the indicator.

### Limits of the study

This study has several limits. 1/ For the medication-based approach, the target populations were identified on the basis of medications prescribed by doctors, not on the basis of refunded medications as it would be in the case of a P4P programme. Unfortunately we did not have access to the Health Insurance reimbursement database. No study today enables us to assess the extent of the gap there may be between medications prescribed and medications bought by the patients and reimbursement by health insurance. We can posit that it is narrow in these populations of patients with chronic diseases who tend to be compliant with their doctor's prescription. 2/ We cannot conclude on the statistical significance (or not) of the difference between the scores according to the mode of identification, as the construction of the indicators was based on different populations. We can only conclude that the difference is of 5% in our sample, and that the confidence interval is not large, especially since the number observations (number of doctors, n = 61) is not big. 3/ Data collection for this study was carried out on a volunteer basis and not in the framework of P4P. Judging by the British case, it is possible that clinical under-coding exists with P4P [7]. This under-coding would tend to reduce the difference between the two types of identification by artificially improving the indicator calculated on the basis of clinical identification. 4/ The correlation between the two search modes in this sample of French practices is strikingly good and probably reflects good use of diagnostic computer entry in these patients who attend on a regular basis for routine monitoring. This may not be generalizable across other conditions.

### Conclusion

Our findings do not enable us to conclude that one of the two identification methods is better than the other. The two approaches yield very similar scores but these scores cover different realities and offer food for thought on the possible usage of these indicators in the framework of P4P programmes. Although it may seem reasonable to use these indicators in order to compare a doctor's activity from one year to the next regardless of the identification mode, provided that it remains the same. On the other hand, using the absolute value of an indicator seems meaningless either for estimating the intrinsic quality of care delivery, or to compare doctors between each other because it depends, among other things, on how it was built.
